# Microbial Shifts After Sleeve Gastrectomy: The Gut–Oral Axis, Periodontal Outcomes, and Competing Oral Risks

**DOI:** 10.3390/biomedicines14040838

**Published:** 2026-04-07

**Authors:** Felicia Gabriela Beresescu, Razvan Marius Ion, Adriana-Stela Crisan, Andrea Bors

**Affiliations:** 1 Faculty of Dental Medicine, George Emil Palade University of Medicine, Pharmacy, Science and Technology of Targu Mureș, 540139 Targu Mureș, Romania; 2 Second Department of Surgery, George Emil Palade University of Medicine, Pharmacy, Science and Technology of Targu Mureș, 540139 Targu Mureș, Romania; 3 Doctoral School of Medicine and Pharmacy, George Emil Palade University of Medicine, Pharmacy, Science and Technology of Targu Mureș, 540139 Targu Mureș, Romania; 4 2nd Department of Surgery, Mureș County Emergency Hospital, 540136 Targu Mureș, Romania; 5 Genetics Department, George Emil Palade University of Medicine, Pharmacy, Science and Technology of Targu Mureș, 540139 Targu Mureș, Romania; 6 Genetics Laboratory, Center for Advanced Medical and Pharmaceutical Research of George Emil Palade University of Medicine, Pharmacy, Science and Technology of Targu Mureș, 540139 Targu Mureș, Romania

**Keywords:** sleeve gastrectomy, bariatric surgery, periodontitis, gut–oral axis, severe obesity, systemic inflammation

## Abstract

**Background**: Severe obesity is associated with chronic low-grade inflammation, dysglycemia, and higher periodontitis risk. Sleeve gastrectomy (SG) is now a dominant bariatric procedure and reliably improves weight and metabolic status yet reported oral and periodontal trajectories after surgery remain heterogeneous. **Objective**: To synthesize SG-centered evidence on periodontal outcomes, oral and gut microbiome remodeling, and mechanistic pathways that may link postoperative physiology to the gut–oral axis. **Methods**: We conducted a structured narrative review guided by SANRA principles using targeted searches of PubMed/MEDLINE, Web of Science, Scopus, and Embase, complemented by citation chaining of key reviews and mechanistic anchor papers; evidence was organized into clinical, oral microbiome, gut microbiome, and mechanistic gut–oral axis streams and interpreted with a pragmatic evidence hierarchy. **Results**: Small prospective SG cohorts suggest bleeding on probing (BOP), gingival indices, and sometimes probing depth (PD) may improve in some patients, particularly alongside weight loss, improved glycemic control, and lower systemic inflammatory burden, whereas clinical attachment level (CAL) and longer-term structural trajectories remain mixed; mixed-procedure syntheses also report early deterioration in some settings. Oral microbiome findings after bariatric surgery are site- and time-dependent, and salivary signals do not necessarily mirror subgingival plaque, whereas gut microbiome remodeling and bile acid signaling changes are more consistently reported and provide plausible but indirect mediator candidates. At the same time, reflux, vomiting, salivary changes, diet patterning, medications, and periodontal care can modify or counteract potential periodontal benefits and may increase competing risks such as caries or erosive tooth wear. **Conclusions**: The SG–gut–oral axis-periodontal pathway is a biologically plausible working hypothesis rather than a proven causal pathway in humans. The present evidence for any periodontal benefit relies mainly on small observational cohorts and is most credibly demonstrated for inflammatory, not structural, endpoints.

## 1. Introduction

Obesity is an escalating, chronic, and relapsing global disease increasingly framed as a multisystem condition with endocrine, immune, and metabolic sequelae rather than excess body mass alone. International analyses show sustained growth in adult obesity prevalence and project further increases, especially in settings with high cardiometabolic burden [1]. Severe obesity concentrates inflammatory and metabolic risk and frequently co-occurs with behavioral and treatment-related exposures that can shape mucosal immunity and microbial ecology.

Metabolic and bariatric surgery is an evidence-based intervention for patients with severe obesity and obesity-related complications, producing clinically meaningful weight loss and improvement in metabolic disease. Current ASMBS/IFSO guidance recommends surgery for patients with body mass index (BMI) ≥ 35 kg/m^2^ regardless of comorbidity status and consideration at lower BMI thresholds when metabolic disease is present [2].

Laparoscopic sleeve gastrectomy (SG) is the most common primary procedure in contemporary bariatric practice in many settings [3,4]. Global registry data confirms the high procedural volume of SG, although regional variation persists [4,5]. This procedural dominance creates an important knowledge gap: SG is performed at scale, but procedure-specific oral, periodontal, and microbiome outcomes are less well characterized than weight-loss and metabolic endpoints.

Periodontal diseases are among the most prevalent oral conditions worldwide. Global Burden of Disease 2021 estimates indicate that periodontal disease affected approximately 1.07 billion people in 2021, with almost 90 million incident cases and substantial non-fatal health loss (about 6.9 million DALYs) [6]. This burden makes periodontitis a key target for integrated prevention and chronic disease management.

Burden differs geographically and socioeconomically and is inversely associated with the sociodemographic index [6]. Contemporary periodontal classification uses staging and grading to describe severity/complexity and the likelihood of progression. For example, stage III, grade C periodontitis reflects advanced destruction with rapid progression potential, which may be influenced by systemic modifiers such as obesity and diabetes. Using a shared staging/grading language improves comparability across studies and supports clinically meaningful risk stratification [7,8,9,10,11].

Robust epidemiologic evidence supports an association between obesity and periodontitis [12]. Meta-analyses across diverse populations and study designs report modest but consistent increases in periodontitis odds among people with obesity [13,14]. Although effect sizes vary, they align with biologically plausible mechanisms in which obesity-related immunometabolic dysregulation elevates inflammatory tone and impairs resolution, potentially facilitating oral dysbiosis and heightened tissue susceptibility.

Host physiology and microbial ecosystems change after metabolic surgery, but reported periodontal trajectories vary across studies [15,16]. Key effect modifiers include baseline periodontal status, diabetes, and smoking. Syntheses that aggregate procedures (SG and Roux-en-Y gastric bypass (RYGB)) report heterogeneous trajectories of bleeding on probing, plaque-related indices, probing depth, and clinical attachment level, with some studies suggesting early postoperative worsening and others later stabilization or improvement [15,16,17,18,19]. Heterogeneity is expected because surgery co-occurs with time-varying exposures that can recalibrate oral ecology, including diet patterning, reflux or vomiting, micronutrient status, antibiotic and acid-suppressive therapy, and access to periodontal care [17,18]. These variables should be captured and modeled explicitly to support more credible causal inference.

Procedure-specific clinical evidence for SG is emerging and may help contextualize heterogeneity in mixed-procedure analyses. Available small prospective SG cohorts suggest that some periodontal inflammatory indices can improve during follow-up in at least some patients, often alongside weight loss, improved glycemic control, and lower systemic inflammatory markers [20,21]. However, most studies use pre–post observational designs, lack contemporaneous controls, and remain vulnerable to residual confounding related to oral hygiene, medications, reflux burden, smoking, and periodontal care [22,23].

While SG offers potential periodontal benefits, these must be interpreted alongside significant competing oral risks. SG-induced changes in reflux symptoms, meal patterning, salivary ecology, micronutrient status, and medication exposure can reshape oral biofilms, potentially influencing caries, erosion, xerostomia, and periodontal measurements [24,25,26]. These considerations argue against treating metabolic surgery as a single uniform oral-health exposure and support procedure-specific interpretation.

SG is associated with gut microbiome remodeling, and procedure-related physiological change may reshape the intestinal ecosystem beyond weight loss alone [27,28,29]. Cohort studies report that both SG and Roux-en-Y gastric bypass (RYGB) can produce rapid gut microbiome shifts [29]. Mechanistic reviews highlight bile acids and their receptors (farnesoid X receptor [FXR] The graphical abstract now color-codes evidence levels, with green indicating comparatively consistent human/postoperative signals, amber indicating indirect or hypothesis-generating mediator links, and red indicating competing oral risks or uncertainty. and G protein-coupled bile acid receptor 1 [TGR5]) enteroendocrine signaling, and microbial bile acid transformations as part of a network linking metabolic surgery to barrier function and systemic immune tone, with plausible downstream relevance to periodontal inflammation [27,28]. Because sequencing data is compositional, pairing relative abundance profiles with quantitative approaches such as quantitative polymerase chain reaction (qPCR) can improve inference about microbial load changes [29,30]. Clinical reports also suggest that systemic inflammatory indices (e.g., CRP) and periodontal inflammatory indices (e.g., bleeding on probing [BOP]) may decrease after surgery, although effect sizes depend on study design, follow-up timing, and perioperative exposures [31,32,33,34].

Taken together, the literature supports an SG-centered working hypothesis: postoperative metabolic improvement, altered gastrointestinal physiology, bile acid signaling, barrier function, and microbial remodeling may collectively reduce periodontal inflammatory activity in some patients; however, the net oral trajectory likely varies according to local ecological pressures, reflux, diet patterning, smoking, diabetes status, and the intensity of professional periodontal care [25,35].

Accordingly, this structured narrative review synthesizes clinical periodontal outcomes, broader oral health outcomes, oral and gut microbiome shifts, and mechanistic evidence relevant to the gut–oral axis. Our aims are to explain heterogeneity in reported periodontal trajectories after metabolic surgery, assess whether SG-associated biological changes provide plausible mediator candidates for periodontal improvement, and define methodological priorities for future longitudinal, controlled, multi-omics studies with parallel oral and gut sampling.

**Figure 1:**
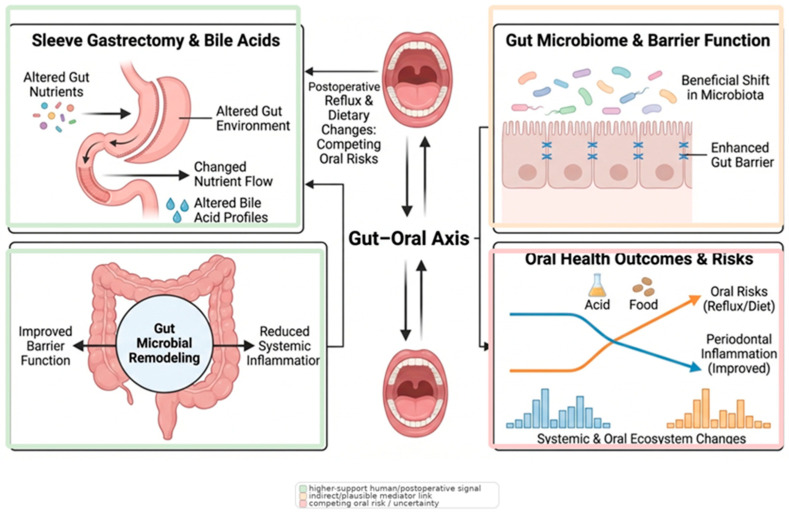
Graphical abstract illustrating hypothesized pathways linking sleeve gastrectomy (SG) to periodontal trajectories via the gut–oral axis; green denotes comparatively consistent hu-man/postoperative signals, amber denotes indirect or plausible mediator links, and red denotes competing oral risks or uncertainty.” created in BioRender. Beresescu, F. G. (https://BioRender.com/gchb4wz, accessed on 28 March 2026) is licensed under CC BY 4.0.

Figure 1 summarizes the proposed SG gut–oral axis, emphasizing that any clinical suggestion for postoperative periodontal surveillance should be interpreted as expert opinion informed by limited observational evidence rather than as a formal guideline. The graphical abstract color-codes evidence levels, with green indicating comparatively consistent human/postoperative signals, amber indicating indirect or hypothesis-generating mediator links, and red indicating competing oral risks or uncertainty. The diagram is conceptual and intended to explain heterogeneity in reported outcomes rather than depict a single established causal chain. Abbreviations: SG, sleeve gastrectomy; FXR, farnesoid X receptor; TGR5, G protein-coupled bile acid receptor 1.

## 2. Methods

This is a structured narrative review, not a systematic review or scoping review. It integrates clinical periodontal findings, oral and gut microbiome evidence, and mechanistic literature relevant to the gut–oral axis in the context of sleeve gastrectomy (SG). Reporting transparency was guided by the Scale for the Assessment of Narrative Review Articles (SANRA), with emphasis on the justification of the topic, description of the literature search, balanced synthesis, and cautious interpretation. Because the goal was explanatory synthesis rather than exhaustive effect estimation, no PRISMA flow diagram or quantitative pooled analysis was undertaken [36,37,38,39].

Targeted searches using predefined search strings were conducted in MEDLINE/PubMed, Web of Science, Scopus, and Embase (database inception through 31 December 2025; last search 31 December 2025). Records were screened for direct relevance to SG, bariatric surgery, periodontitis, oral microbiome, gut microbiome, and gut–oral axis mechanisms; citation chaining from high-impact reviews and anchor mechanistic papers was used to capture studies that informed causal interpretation, effect modifiers, or competing oral risks.

Search concepts combined controlled vocabulary and keywords for surgical exposures (SG, vertical sleeve gastrectomy, bariatric/metabolic surgery), periodontal outcomes (periodontitis, bleeding on probing, probing depth, clinical attachment loss, plaque indices), broader oral outcomes (caries, dental erosion, xerostomia, halitosis, saliva), microbiome endpoints (microbiome, microbiota, 16S, metagenomics, metabolomics), and mediator terms (bile acids, barrier function, endotoxemia, gut–oral axis). A representative PubMed syntax was: ((“sleeve gastrectomy” OR “vertical sleeve gastrectomy” OR bariatric surgery OR metabolic surgery) AND (periodontitis OR periodontal OR “bleeding on probing” OR “probing depth” OR “clinical attachment loss” OR caries OR erosion OR xerostomia OR halitosis OR saliva OR microbiome OR microbiota OR stool OR gut OR oral) AND (“bile acid*” OR endotoxemia OR barrier OR “gut–oral axis”)).

Four evidence streams were defined as follows:

1. Clinical periodontal outcomes: Human adults (≥18 years) undergoing SG with defined periodontal measures and ≥3 months of follow-up; studies were considered SG-specific if SG participants were analyzed separately (stand-alone SG cohort or SG subgroup analysis).
2. Oral microbiome: Human studies reporting postoperative changes in salivary, supragingival, and/or subgingival microbial communities after SG (or mixed bariatric cohorts when SG-specific reporting was unavailable).
3. Gut microbiome: Human studies (including systematic reviews/meta-analyses) reporting fecal or intestinal microbiome composition and/or diversity after SG (or bariatric surgery cohorts including SG).
4. Mechanistic gut–oral axis: Animal, ex vivo, and human translational studies addressing oral-to-gut microbial transmission, barrier/endotoxemia pathways, immune programming, and metabolite signaling relevant to periodontal inflammation.

Periodontal outcomes were grouped as inflammation-related indices (e.g., Plaque Index [Silness-Loe], Gingival Index [Loe-Silness], Ainamo-Bay bleeding metrics, bleeding on probing, and O’Leary Plaque Control Record) and structural measures (probing depth and clinical attachment level), recorded according to the original study definitions. Where broader oral outcomes were discussed, we extracted study-specific measures for caries (e.g., DMFT/DMFS or comparable clinical reporting), dental erosion, halitosis (organoleptic or volatile sulfur compound-based assessment when reported), xerostomia/salivary change, and reflux-related oral symptoms [40,41,42].

For temporal interpretation, postoperative observations were grouped pragmatically into early (<3 months), intermediate (3–12 months), and later (>12 months) follow-up. This categorization was used because transient early postoperative changes (diet phases, vomiting/reflux burden, antibiotics, acid suppression, and rapid weight loss) may differ materially from later stabilization windows.

Study quality considerations informed interpretation rather than serving as exclusion criteria. For randomized trials, RoB 2 domains were used as interpretive lenses; for nonrandomized clinical studies, we noted key risks including pre–post designs without contemporaneous controls, inconsistent periodontal calibration, heterogeneous follow-up, incomplete confounder capture, and selective reporting [43,44,45,46,47,48,49]. To improve transparency, we described the resulting evidence in pragmatic hierarchy terms: SG-specific prospective cohorts and mixed-procedure systematic reviews/meta-analyses were treated as low-to-moderate clinical evidence because they ultimately rest on observational data, whereas animal, ex vivo, and translational gut–oral axis studies were treated as indirect mechanistic support rather than proof of human mediation.

The synthesis was conducted narratively by triangulating the four evidence streams. Interpretive weight was preferentially assigned to prospective SG cohorts with standardized periodontal examinations and clearly described perioperative exposures, followed by mixed-procedure syntheses used mainly to contextualize heterogeneity, while translational papers linking microbial remodeling with plausible mediator pathways were interpreted as hypothesis-supportive but indirect. Table 1 provides a structured evidence map, including sample size, baseline periodontal context, diabetes/smoking information when reported, and the role of each study in the evidence hierarchy. As illustrated in Figure 1, better-supported links (solid arrows) are distinguished from plausible but not directly demonstrated SG–periodontal mediation paths (dashed arrows).

## 3. Microbial Remodeling After Sleeve Gastrectomy and Its Plausible Links to Periodontal Improvement

### 3.1. Conceptual Rationale: Obesity, Dysbiosis, and Host Susceptibility

Severe obesity is increasingly conceptualized as an immunometabolic disease, with hypertrophic adipose tissue functioning as an endocrine–immune organ that sustains chronic low-grade inflammation and metabolic dysfunction [52]. This systemic milieu may lower the threshold for persistent periodontal inflammation and may also influence gut and oral microbial ecology [53]. Furthermore, host genetic factors may play a crucial role in individual susceptibility to both obesity-related inflammatory responses and periodontal disease progression, warranting consideration in future multi-omics investigations. In bariatric cohorts, microbial and metabolic remodeling has been reported across gut and oral compartments, and bile acid–microbiota interactions have been linked with inter-individual variability in weight-loss and metabolic responses [54,55,56,57]. Although most human evidence remains associative, these observations support a framework in which surgery-related ecological perturbations and microbially derived metabolites contribute to variability in downstream oral outcomes.

In this context, obesity-related immunometabolic dysregulation may increase periodontal susceptibility by elevating baseline inflammatory mediators, impairing immune function, and altering the local tissue microenvironment, thereby influencing microbial selection and resolution capacity [53,58]. Oral microbial communities also appear to shift after bariatric surgery, but reported signals are typically smaller and more heterogeneous than those observed in the gut. Longitudinal salivary profiling indicates substantial inter-individual variability, likely reflecting differences in baseline periodontal status and local ecological pressures (dietary adherence, oral hygiene, reflux burden, medication exposure, and perioperative dental care). After SG, postoperative dietary changes (food texture, meal timing/frequency, and macronutrient composition) can alter oral pH and substrate availability, creating selection pressures that may reshape biofilm ecology [50].

**Figure 2:**
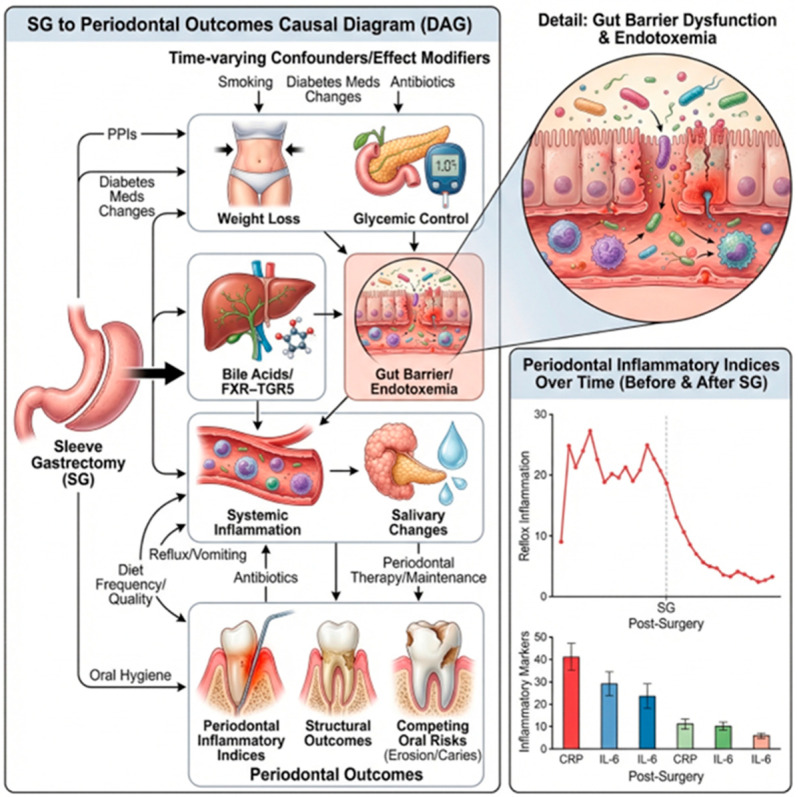
Conceptual framework summarizing effect modifiers, mediator candidates, and com-peting risks that may shape periodontal outcomes after sleeve gastrectomy (SG). Solid arrows indicate comparatively better-supported bariatric or experimental links; dashed arrows indicate plausible but still indirect SG–periodontal mediation.” created in BioRender. Beresescu, F. G. (https://BioRender.com/4wde4mj, accessed on 28 March 2026) is licensed under CC BY 4.0.

A conceptual framework summarizing effect modifiers, mediator candidates, and competing risks that may shape periodontal outcomes after sleeve gastrectomy (SG) is shown in Figure 2. The scheme differentiates comparatively better-supported links (solid arrows) from plausible but not directly demonstrated SG–periodontal mediation paths (dashed arrows). The framework distinguishes (i) systemic pathways (metabolic improvement, reduced low-grade inflammation, altered bile acid–microbiome signaling, and barrier/immune modulation) from (ii) local oral habitat drivers (reflux/vomiting, diet frequency/texture, salivary flow and buffering, antibiotic/proton pump inhibitor exposure, and oral hygiene/periodontal maintenance). Periodontal outcomes are grouped as inflammation-related indices (e.g., bleeding on probing/gingival indices) that may change earlier and structural endpoints (probing depth/clinical attachment level) that typically evolve more slowly and are sensitive to measurement standardization and periodontal therapy. Key confounders (smoking, diabetes therapy changes, baseline periodontal phenotype, and professional periodontal care) can produce heterogeneous postoperative trajectories. Abbreviations: SG, sleeve gastrectomy; BOP, bleeding on probing; PD, probing depth; CAL, clinical attachment level; PPI, proton pump inhibitor; NSPT, non-surgical periodontal therapy.

### 3.2. Oral Microbiome Remodeling After Bariatric Surgery

Studies of saliva, crevicular fluid, and periodontal-adjacent niches suggest that bariatric surgery is followed by changes in oral microbial community structure and inflammatory biomarkers, although the direction of change is strongly site-, time-, and exposure-dependent [30,51,59]. Some cohorts report ecological shifts without parallel short-term clinical periodontal improvement, implying that oral microbial remodeling and periodontal phenotypes may move on different time scales [59,60]. A recent scoping review likewise concluded that postoperative oral microbiome changes are likely but heterogeneous, with interpretation shaped by sampling niche, baseline periodontal status, diet, reflux, salivary changes, and oral hygiene [61].

### 3.3. Periodontal Clinical Outcomes After SG and Mixed-Procedure Syntheses

SG is particularly relevant because its effects extend beyond gastric restriction to rapid changes in insulin sensitivity, glycemia, and systemic inflammatory tone [62,63]. Meta-analyses across bariatric procedures report postoperative reductions in C-reactive protein (CRP) and interleukin-6 (IL-6), but SG-specific effect sizes remain less certain and depend on baseline phenotype and follow-up timing [33,64]. From a periodontal perspective, downshifting systemic inflammation could reduce gingival hyper-responsiveness to biofilm challenge and help explain why bleeding or gingival indices may improve earlier than probing depth or clinical attachment level, especially when structural measures are assessed over short follow-up windows without standardized periodontal therapy [7,8,15,16,17,18,19].

The periodontal outcome literature remains heterogeneous when procedures are grouped together. Systematic reviews and meta-analyses report variable effects on probing depth, clinical attachment level, bleeding on probing, and plaque-related indices, with some studies showing early deterioration and others later stabilization or improvement [15,16,17,18,19]. Much of this inconsistency likely reflects differences in outcome definitions, follow-up windows, and confounder capture, which are addressed separately in Section 3.5.

SG-specific prospective evidence, while still limited, helps clarify procedure-specific periodontal trajectories. A 1-year nonrandomized study in patients undergoing SG, including individuals with and without type 2 diabetes, reported improvement in plaque/bleeding indices during follow-up alongside weight loss, better glycemic control, and lower inflammatory biomarkers, but probing depth and clinical attachment level remained comparatively stable [20]. Another prospective SG cohort likewise described postoperative improvement in plaque, gingival, and bleeding indices over early follow-up, whereas probing depth and clinical attachment level did not significantly change [21]. These findings do not establish causality and should be read as small uncontrolled signals rather than definitive proof of periodontal benefit; they mainly support the possibility that inflammation-related trajectories after SG may differ from the early deterioration reported in some mixed-procedure analyses or may become more favorable after postoperative physiology and behaviors stabilize [15,16,17,18,19].

### 3.4. Gut Microbiome Remodeling and Mediator Pathways

Gut microbiome remodeling is a plausible mediator of SG-associated metabolic and inflammatory change. Meta-analyses and longitudinal studies report postoperative shifts in gut microbial diversity and composition after SG and Roux-en-Y gastric bypass, consistent with surgery-related physiological changes reshaping the intestinal ecosystem beyond weight loss alone [29,65,66]. Some studies link postoperative microbiome variation with host metabolic readouts, and experimental work supports causal microbiome contributions to selected metabolic phenotypes [67,68]. Bile acids are a particularly important signaling interface connecting metabolic surgery, the microbiome, and host immune–metabolic pathways via FXR and TGR5. Paired microbiome–metabolomics designs may therefore help identify taxa–metabolite patterns that can be tested as candidate mediators in future SG cohorts [69,70].

Studies generally indicate that while the oral microbiome can change after bariatric surgery, interpretation remains difficult because results depend strongly on sampling site and follow-up interval. Longitudinal salivary studies suggest that oral community composition can shift postoperatively, yet inter-individual variability is substantial and likely reflects local ecological pressures such as diet, pH, saliva composition, reflux, and oral hygiene [50]. This distinction is methodologically important: saliva integrates shed organisms and host products from the whole mouth, whereas subgingival plaque samples the periodontal pocket microenvironment more directly. The two niches need not move in parallel after SG. Systematic reviews likewise conclude that postoperative oral microbiome changes are likely but heterogeneous, with interpretation shaped by sampling niche, baseline periodontal status, diet, reflux, salivary changes, and oral hygiene [61,71]. The main sources of heterogeneity are synthesized in Section 3.5 and Table 1.

Some datasets indicate that community profiles shift toward states less associated with periodontitis; however, other datasets show enrichment of taxa linked to periodontitis, caries, or halitosis after surgery and interpret this as persistent or redistributed oral dysbiosis despite metabolic improvement [61,72,73,74]. The direction of change likely depends on sampling niche and baseline periodontal status, as well as time since surgery, antibiotic and acid-suppression exposure, reflux burden, salivary changes, and changes in dietary substrate availability, all of which can alter local ecological selection pressures in the oral cavity [25,50,61,71,72,73]. From a periodontal perspective, systemic metabolic improvement after SG may reduce inflammatory responsiveness, but local postoperative pressures may concurrently favor cariogenic or aciduric shifts unless preventive behaviors and professional care are maintained [25]. Accordingly, salivary improvement should not be over-read as direct evidence of subgingival or clinical periodontal recovery.

Table 1 summarizes the main evidence streams contributing to the Section 3 synthesis, and now also records sample size, baseline periodontal context, diabetes/smoking data when reported, and the principal caveats relevant to interpretation.

### 3.5. Outcome Definitions, Follow-Up Windows, and Time-Varying Confounding

Apparent inconsistency across studies partly reflects the fact that not all oral endpoints capture the same biological process or time scale. Inflammatory indices such as bleeding on probing, gingival indices, or plaque scores can respond within weeks to shifts in host inflammatory tone, plaque control, or perioperative dental care, whereas probing depth and clinical attachment level require longer observation and are more sensitive to baseline destruction, examiner calibration, and adjunctive periodontal therapy. Likewise, broader oral endpoints such as caries, dental erosion, halitosis, xerostomia, and salivary flow reflect different mechanisms and should not be interpreted as interchangeable with periodontitis improvement.

Follow-up timing is also crucial. For this review, we interpret early postoperative changes as <3 months, intermediate changes as 3–12 months, and later changes as >12 months. Early windows may overrepresent diet phases, vomiting/reflux, antibiotic exposure, acid suppression, and abrupt behavioral change, whereas later windows are more informative for stabilized metabolic and ecological adaptation. This temporal distinction helps reconcile reports of early worsening or dysbiosis with later stabilization or improvement.

Several confounders can meaningfully alter periodontal trajectories after SG: smoking status, baseline diabetes and changes in antidiabetic therapy, perioperative antibiotics and proton pump inhibitors, dietary frequency and texture, oral hygiene behavior, professional periodontal treatment (including non-surgical periodontal therapy [NSPT]), and access to follow-up care. Because many of these exposures change over time and can also influence both the microbiome and periodontal indices, studies that fail to capture them are vulnerable to misattributing postoperative changes to SG itself. Procedure type matters as well: SG is generally less malabsorptive but more reflux-prone than Roux-en-Y gastric bypass, so the balance between systemic metabolic benefit and local oral acid exposure may differ by operation [25].

### 3.6. Evidence for Oral–Gut Trafficking and Immune Programming

These clinical and ecological observations can be integrated within a gut–oral axis framework. Experimental evidence characterizes some oral organisms as able, under certain dysbiotic conditions, to ectopically colonize the intestine and induce proinflammatory immune responses, supporting the feasibility of oral-to-gut microbial transmission with immunologic consequences [74]. Other work describes an intramucosal connection in which oral pathobionts expand during periodontal inflammation, translocate to the gut, and trigger intestinal inflammation in susceptible hosts, supporting the plausibility that oral dysbiosis can modulate distal mucosal immunity and systemic inflammatory tone [75,76]. Mechanistic studies also show that key periodontal pathogens (e.g., Porphyromonas gingivalis) can aggravate colitis through gut microbiota- and metabolite-dependent immune mechanisms involving Th17/Treg balance, providing a strong example of microbiota-mediated oral–gut crosstalk [77,78,79].

Human evidence on oral-to-gut microbial trafficking in bariatric populations is necessarily less direct than experimental models. Nonetheless, studies of patients undergoing bariatric surgery have reported periodontal pathogens and Helicobacter pylori in oral and gastric specimens, suggesting that microbial niches across the oral and upper gastrointestinal tract may be linked and can shift after surgery, consistent with altered niche selection pressures in the postoperative stomach and gut [80,81]. From this perspective, SG could plausibly influence periodontal outcomes not only through reduced adiposity and systemic inflammatory downshifting, but also through altered gastrointestinal ecology and host-microbe signaling.

While these pathways are plausible modulators of oral immunity and microbial community stability, direct human evidence showing that SG-induced change in specific mediators causes periodontal improvement remains limited and warrants cautious interpretation [29,69,71,75,77]. The strongest support for oral–gut trafficking still comes from experimental models, whereas bariatric human data are indirect and mostly associative.

### 3.7. Competing Oral Risks and Clinical Implications

At the same time, the same gut–oral axis implies that postoperative exposures can also push oral outcomes in an unfavorable direction. Erosive tooth wear is promoted by acidic pH exposure from gastroesophageal reflux and vomiting, both of which are reported after bariatric procedures, including SG [25]. Registry-based comparisons of dental outcomes after gastric bypass and SG indicate that oral healthcare utilization and intervention rates may differ by procedure type, supporting procedure-specific oral health surveillance rather than assuming a single “bariatric surgery” effect [24]. Salivary flow and composition can also change after bariatric surgery; systematic reviews report heterogeneous findings, but saliva is clinically important for buffering, remineralization, and oral biofilm ecological stability [82]. Changes in salivary microbial markers have also been reported after surgery [83]. Together, these exposures may increase caries or erosion risk even when periodontal inflammatory indices improve, helping to explain why improvements in periodontitis can coexist with signals of oral dysbiosis and why some indices may transiently worsen in early postoperative windows [25].

Taken together, the available clinical and microbial evidence does not support a uniform direction of periodontal change after SG. Inflammation-related indices may improve in some cohorts in parallel with weight loss, improved glycemic control, and reduced systemic inflammatory burden, whereas structural outcomes evolve more slowly and are harder to interpret without standardized periodontal therapy and examiner calibration. A pragmatic graded clinical approach is therefore more defensible than universal postoperative surveillance: high priority—baseline oral examination, oral-hygiene reinforcement, and erosion/caries prevention in all SG candidates; targeted periodontal monitoring—reassessment of BOP, probing depth, and risk factors in patients with established periodontitis (especially stage III/grade C), diabetes, smoking, reflux, or recent periodontal therapy; lower priority—routine intensified periodontal follow-up for otherwise low-risk patients, which is currently preventive rather than evidence-based.

## 4. Limitations of the Current Evidence Base

The current evidence base linking sleeve gastrectomy to periodontal improvement through microbial remodeling is suggestive but not definitive.

Limitations arise at several inferential levels within the primary literature, including heterogeneity in clinical periodontal measurement, challenges with time-varying exposure confounding, inconsistencies in microbiome sampling and reporting, and the absence of direct mediation analyses. Furthermore, as a narrative review, our synthesis provides a qualitative overview rather than pooled effect estimation.

As outlined in Section 3.5, confounding and bias are substantial because bariatric surgery co-occurs with multiple exposures that can independently affect oral ecology and periodontal inflammation, including antibiotics, acid suppression, diet patterning, reflux and vomiting, smoking behavior, diabetes pharmacotherapy and periodontal care. These factors are often incompletely captured or treated as static rather than time-varying, limiting causal attribution.

The evidence on the microbiome offers further limitations for causal inference. There are few studies, which on average have low power, that have investigated the oral microbiome after bariatric surgery. Further, there are significant differences in sampling niche, sequencing approach, and analytic pipelines, making generalization of outcomes difficult and contributing to the mixed findings across studies and reviews [50,61,71,72,73]. The specificity of sampling sites must be considered. Salivary signals often reflect the ecological or dietary or salivary changes in a whole-mouth setting, while subgingival plaque is more closely linked to periodontitis pathogenesis. Relying solely on salivary signals will therefore mean that a study captures ecological change but not the periodontal niche directly. Research on the gut microbiome faces similar challenges, where fecal samples provide an easy proxy, but might not fully represent the closer mucosa-associated communities that more directly interact with host immunity. Understanding both oral and gut microbiome studies is made difficult by missing key metadata, including detailed characterization of diet, exposures to antibiotics and proton pump inhibitors, oral hygiene behaviors, reflux symptoms, and sample collection and storage information. Reporting frameworks like STORMS and the Genomic Standards Consortium minimum-information standards specify these variables as critical for reproducibility and cross-study comparability; consistent implementation of the same would materially strengthen the evidence for the bariatric gut–oral axis [84,85].

The microbiome evidence has further limitations for causal inference. Few adequately powered studies have evaluated the oral microbiome after bariatric surgery, and existing reports differ in sampling niche, sequencing approach, and analytic pipelines, making generalization difficult and contributing to mixed findings across studies and reviews [50,61,71,72,73]. Site specificity matters: saliva reflects a whole-mouth composite strongly influenced by salivary flow, reflux, diet, and oral hygiene, whereas subgingival plaque more directly captures the periodontal pocket habitat. Accordingly, discordance between salivary and subgingival signals should be expected rather than treated as contradictory.

Analytic constraints also require careful attention. Microbiome data are compositional, and relative abundance changes do not directly equate to absolute abundance changes without complementary quantification; this property makes simple ‘enrichment’ narratives vulnerable to misinterpretation when the denominator shifts or when total microbial loads change across time points or treatment phases [86]. Preprocessing choices can also affect downstream conclusions. Rarefaction has been criticized as statistically inadmissible in many contexts because it discards data and can reduce power, and more recent comparative work emphasizes that normalization and differential abundance strategies should be selected based on data characteristics rather than applied uniformly [87,88]. In future SG studies, compositionality-aware, bias-corrected differential-abundance approaches (for example, analysis of compositions of microbiomes with bias correction, ANCOM-BC) should be paired, when feasible, with absolute quantification such as quantitative polymerase chain reaction (qPCR), flow cytometry, or fluorescence in situ hybridization. In the bariatric context, perioperative medication exposures are especially salient confounders. Antibiotics can induce large, individualized, and incompletely reversible disruptions of the gut microbiome [89], while proton pump inhibitors can shift both gut and potentially oral-source trafficking signals [90,91]. Without explicit capture and analytic control, these signatures can be misattributed to surgery.

These limitations collectively constrain the ability to determine whether gut microbiome remodeling mediates periodontal improvement after sleeve gastrectomy. Most human studies use pre–post observational designs, often without robust control groups or time-resolved mediator measurements required for credible mediation analysis [66]. Even when temporal sampling exists, the hypothesized pathway likely includes multiple interacting mediators, including bile acid profiles, short-chain fatty acids, gut permeability or endotoxemia markers, and systemic inflammatory cytokines, and is plausibly bidirectional because periodontal inflammation can itself contribute to systemic inflammatory load and may influence gut ecology through oral-to-gut microbial seeding. Mechanistic studies demonstrating oral-to-gut colonization and immune imprinting establish feasibility and directionality in experimental systems, but translating these mechanisms to sleeve gastrectomy cohorts requires direct human measurement of proposed mediators and parallel sampling of gut and periodontal niches in longitudinal designs [69,74,75,77,84,85,92,93,94,95]. Without such data, mechanistic claims remain plausible but incompletely evidenced in the SG setting.

In practical terms, the main limitations are heterogeneity of sampling niches and timing, differences in sequencing platforms and bioinformatics pipelines, lack of standardized clinical oral outcomes across studies, and probable publication or selection bias favoring positive or mechanistically striking findings. These limitations reduce cross-study comparability and preclude strong causal or quantitative inference.

## 5. Future Directions

Future research should prioritize designs capable of moving from plausibility to causal inference. Periodontal outcomes should be measured with standardized full-mouth examinations, including full-mouth probing depth, clinical attachment level, and bleeding on probing at six sites per tooth, performed by clearly calibrated examiners with explicit reporting of inter- and intra-examiner reliability [7,8,96]. Studies should also separate preoperative diet phases from surgery-specific effects and sample early perturbation windows and later steady states. A practical minimum schedule would include baseline before the preoperative diet if feasible, a post-diet/preoperative sample, an early postoperative window (about 1–2 weeks), and follow-up at roughly 3 months and 6–12 months, because microbial and inflammatory dynamics are unlikely to be constant across these phases [29,66]. Paired stool, saliva, and subgingival plaque sampling, together with metabolomics and barrier/endotoxemia markers, would allow direct testing of candidate mediation pathways.

**Figure 3:**
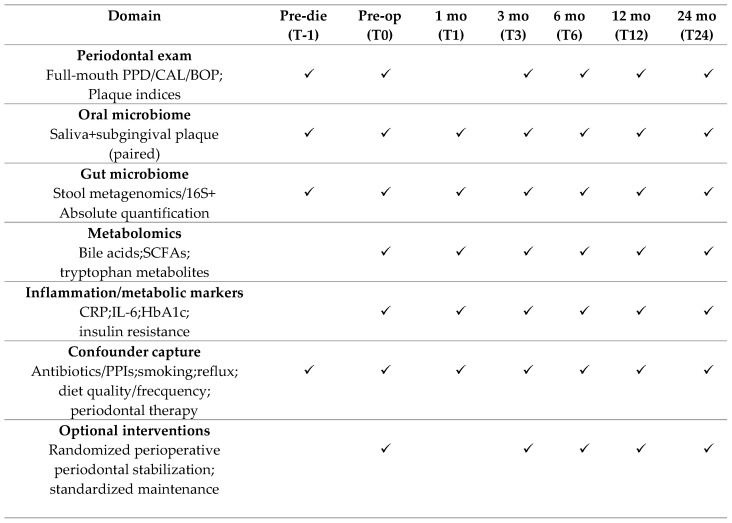
Recommended longitudinal study design to test gut–oral axis mediation after sleeve gastrectomy.

Figure 3 presents a proposed longitudinal framework for mechanistic and mediation-oriented SG studies, incorporating paired gut–oral microbiome sampling, absolute quantification alongside sequencing, metabolomics, standardized periodontal phenotyping, and explicit capture of key confounders.

Studies incorporating standardized periodontal stabilization are likely to be especially informative for translation. Randomizing or otherwise standardizing perioperative non-surgical periodontal therapy (NSP) maintenance intensity and oral-hygiene reinforcement could clarify whether structured dental care amplifies any inflammatory periodontal benefit and reduces postoperative oral dysbiosis under a shared background of metabolic improvement [34,42]. Parallel attention to nutritional follow-up and supplementation adherence is also warranted because micronutrient perturbations may influence oral tissues, host immunity, and the interpretation of oral outcomes after SG [97,98,99,100,101].

Future progress should also prioritize longitudinal designs explicitly powered for mediation testing and supported by modern microbiome reporting standards. Confounder capture should be elevated from an afterthought to a design principle, with explicit recording of antibiotics, proton pump inhibitors, smoking, reflux symptoms, diabetes therapies, diet quality and frequency, oral hygiene behaviors, and exposure to periodontal therapy [92,102]. Microbiome analysis should respect compositionality and report absolute or semi-quantitative complements, when possible, while differential-abundance testing should favor bias-aware approaches rather than rarefaction-dependent workflows [86,87,88]. For gut–oral trafficking questions, paired oral, gastric, and stool sampling with strain-resolved or source-tracking analyses would be more informative than isolated saliva profiling.

Clinically, the current evidence supports a multidisciplinary approach to SG that includes oral-health awareness. As outlined in Section 3.7, a reasonable risk-based approach is baseline oral examination and erosion/caries counseling for all SG candidates, with BOP/probing depth reassessment prioritized for patients with pre-existing periodontitis, diabetes, smoking, reflux, or recent periodontal therapy. More intensive routine surveillance in otherwise low-risk patients should be regarded as preventive rather than as a proven necessity.

## 6. Conclusions

Sleeve gastrectomy is accompanied by metabolic, inflammatory, and microbial remodeling that provides a biologically plausible context for changes in periodontal health. The most defensible conclusion is not that SG uniformly improves periodontitis, but that small observational SG cohorts suggest possible improvement in inflammation-related indices in some patients, whereas structural periodontal endpoints and broader oral trajectories remain heterogeneous and strongly influenced by follow-up window, local postoperative exposures, and professional dental care. Gut microbiome remodeling and oral–gut trafficking remain plausible mediator frameworks, yet the human evidence for mediation is indirect and insufficient to establish causality. Therefore, postoperative periodontal monitoring should currently be described as risk-based expert opinion, not formal guideline-level evidence.

## Figures and Tables

**Table 1:** Summary list of studies presenting the main lines of evidence.

Study (Type)	n	Surgery/Niche	Baseline PD/BOP or Periodontal Context	Diabetes/Smokers	Main Finding	Evidence Role/Key Caveat
Čolak et al., 2021 [15]; Fontanille et al., 2018 [16]; de Souza et al., 2018 [17]; Dos Santos et al., 2019 [18]; Ferraz et al., 2023 [19] (systematic reviews/meta-analyses)	4–9 studies; 250–886 bariatric pts	Mixed bariatric (SG/RYGB/others); Periodontal exams	Mixed case definitions and follow-up windows; PD/CAL/BOP/P variably reported	Rarely stratified; smoking and diabetes incompletely captured.	Early worsening at 6 months reported in several syntheses; later outcomes mixed or inconsistent	Observational synthesis; SG-specific effect unresolved
Bi et al., 2024 [20] (prospective real-world cohort)	106 (57T2D, 49 non-T2D)	SG; full-mouth periodontal indices + inflammatory markers	PD 4.10 vs. 3.68 mm; CAL 2.04 vs. 1.69; BI 2.67 vs. 2.12	53.8% T2D; active smokers excluded	Plaque and bleeding indices improved to 12 months; PD/CAL largely unchanged; hs-CRP and IL-6 fell.	SG-specific favorable inflammatory trend; uncontrolled observational design
Alpan et al., 2024 [21] (prospective cohort)	54	SG; clinical periodontal indices+metabolic markers	PD 2.67 mm; CAL 3.16 mm; BOP 76.8%	40.7% diabetes; 40.7% smokers	PI/GI/BOP improved at 6 months; PD/CAL did not significantly change	Shirt-term inflammatory improvement; smoking/diabetes mix may confound
Džunková et al., 2020 [50] (longitudinal microbiome study)	35 total; SG subset n = 3	Mixed bariatric; saliva	No standardized periodontal phenotype; oral hygiene not monitored at post-op sampling	NR	Large inter-individual salivary shifts after surgery	Whole-mouth signal; not a surrogate for subgingival plaque
Balogh et al., 2020 [51] (follow-up microbiology study)	17 post-BS + controls	Mixed bariatric; crevicular gingival fluid	Uninflamed periodontium; Good oral hygiene	Non-smokers; diabetes NR	Local microflora changed after surgery; Candida/Prevotella shifts; periodontitis did not develop	Niche-specific local signal; baseline susceptibility matters

Notes: n = participants when available; synthesis rows report study/participant ranges or grouped evidence streams. NR = not reported; PD = probing depth; CAL = clinical attachment level; BOP = bleeding on probing; BI = bleeding index; SG = sleeve gastrectomy; RYGB = Roux-en-Y gastric bypass. Diabetes/smoking fields are shown when reported or when design restrictions were explicit.

## Data Availability

No new data were created or analyzed in this study.

## Abbreviations

The following abbreviations are used in this manuscript:

16S	16S ribosomal RNA (gene sequencing marker)
ASMBS	American Society for Metabolic and Bariatric Surgery
BMI	Body mass index
BOP	Bleeding on probing
CAL	Clinical attachment level
CRP	C-reactive protein
DALYs	Disability-adjusted life years
FXR	Farnesoid X receptor
HOMD	Human Oral Microbiome Database
IFSO	International Federation for the Surgery of Obesity and Metabolic Disorders
IL-6	Interleukin-6
MEDLINE	Medical Literature Analysis and Retrieval System Online
NSPT	Non-surgical periodontal therapy
PD	Probing depth
PPI	Proton pump inhibitor
PPIs	Proton pump inhibitors
QIIME 2	Quantitative Insights Into Microbial Ecology 2
ROBINS-I	Risk Of Bias In Non-randomized Studies of Interventions
RoB 2	Risk of Bias 2 (tool for randomized trials)
RYGB	Roux-en-Y gastric bypass
SANRA	Scale for the Assessment of Narrative Review Articles
SG	Sleeve gastrectomy
SILVA	SILVA ribosomal RNA gene database (taxonomy reference)
STORMS	Strengthening The Organization and Reporting of Microbiome Studies (checklist)
STROBE	Strengthening the Reporting of Observational Studies in Epidemiology
TGR5	G protein-coupled bile acid receptor 1
Th17	T helper 17 (cells)
Treg	Regulatory T (cells)
qPCR	Quantitative polymerase chain reaction
